# Blood loss quantification during major abdominal surgery: prospective observational cohort study

**DOI:** 10.1186/s12893-023-02288-w

**Published:** 2024-01-02

**Authors:** Ján Zajak, Jiří Páral, Miroslav Sirový, Šárka Odložilová, Kateřina Vinklerová, Petr Lochman, Filip Čečka

**Affiliations:** 1 Department of Surgery, University Hospital Hradec Kralove, Sokolská 581, Hradec Králové, 500 02 Czech Republic; 2 Department of Military Surgery, Faculty of Military Health Sciences, University of Defence, Hradec Kralove, Czech Republic

**Keywords:** Major abdominal surgery, Liver resection, Pancreaticoduodenectomy, Estimated blood loss, Spectrophotometric measurement

## Abstract

**Background:**

Blood loss during major abdominal surgery is an essential parameter in the evaluation of strategies aimed at reducing perioperative bleeding. However, blood loss quantification remains unreliable and inaccurate. The aim of this study was to compare several methods of blood loss quantification—visual estimation by surgeon and anesthesiologist, the gravimetric method, the calculation method with spectrophotometric measurement. The spectrophotometric measurement is considered as the most accurate method.

**Methods:**

The study was designed as a prospective observational cohort single-center study. We analyzed 61 patients who underwent elective liver or pancreatic resection. The anesthesiologists’ and surgeons' estimate of blood loss was based on a visual assessment. The gravimetric method was based on weighing the suction canister and surgical drapes before and after use. The basis of calculation method was anthropometric and hematological parameters, we used López-Picado's formula. The spectrophotometric method was based on the spectrophotometric determination of hemoglobin mass in the lost blood. We compared the methods using paired t-test, non-parametric Wilcoxon test and Bland–Altman analysis.

**Results:**

Visual estimation by surgeons and anesthesiologists, gravimetric measurement, and calculation method were significantly different from spectrophotometric measurement at the significance level α = 0.05. All methods overestimated blood loss which was measured by spectrophotometric method. The estimate by surgeons was the closest to the spectrophotometric measurement, difference 68.7 ml (95% confidence interval [CI]: -129.3–-8.2).

**Conclusions:**

We conclude that the estimate of blood loss by surgeons and anesthesiologists, as well as gravimetric method and calculation method are all significantly inaccurate in real surgical setting. We did not confirm the commonly accepted assumption that surgeons underestimate the blood loss.

**Trial registration:**

The study was registered under the title " Blood Loss Quantification During Major Abdominal Surgery" at ClinicalTrials.gov with the registration number NCT05316649. Date of the first registration was 20/3/2022.

## Introduction

Blood loss estimate is an important parameter of quality of the surgical procedure, along with postoperative morbidity and mortality, radicality of the surgical resection, number of retrieved lymph nodes, duration of the surgical procedure, duration of the hospital stay, or some other parameters (e.g. postoperative pancreatic fistula in case of pancreatic resections). In addition, intraoperative blood loss is an essential component of many scoring systems used to predict outcomes such as morbidity, mortality, and readmission rates. Quantified blood loss also plays a key role in blood transfusion decisions, along with other information such as hemoglobin values and individual transfusion triggers. Negative impact of intraoperative blood loss on outcomes has been well characterized in many studies. However, blood loss estimation remains unreliable and inaccurate [1–5]. Therefore, it is very important to have more reliable methods to measure the blood loss. The methods used to estimate or to measure blood loss are as follows: visual estimation, gravimetric method, direct measurement, photometry, calculation methods, colorimetric analysis, and miscellaneous [6, 7].

Visual estimation of blood loss by surgeon and anesthesiologist is still the standard method of choice in most institutions [8]. Irrigation fluids, lymph, bile, serum, ascites, urine, and other fluids often combine with lost blood, but do not alter its appearance to an extent that is typically appreciable visually, which can affect estimated blood loss [9]. Visual estimation of operative blood loss is unreliable and inaccurate. No provider specialty, level of experience, or self-assessment of ability is associated with improved estimation [10].

The gravimetric method was first described by Wangensteen and depends on weighing surgical sponges before and after surgical use [11]. Estimated blood loss is determined by assessing the weight difference before and after use, with every gram of weight equivocal to 1 mL of blood loss [12]. This method is easy but neither precise nor accurate [13], especially with increased dilution by rinsing fluid [14, 15].

Direct measurement of blood loss is a simple and long-established method that is mainly used in the field of obstetrics [6]. A calibrated collection bag with a scale is designed for vaginal deliveries. Current blood loss can be immediately read from the scale. Study results show significant deviations from real blood loss [16, 17].

Spectrophotometry is the most precise, but also the most expensive method and the most complex method to measure the blood loss [7]. Hemoglobin mass loss is assessed in the lost blood using the spectrophotometric method. Spectrophotometric measuring of hemoglobincyanide is the reference method for hemoglobinometry in human blood recommended by the International Council for Standardization in Hematology since 1967 (ICSH) [18].

Calculation Method is based on anthropometric and hematological parameters. There are several mathematical formulas and they have been modified over time: Liu, Mercuriali, Bourke, Ward, Gross, Lisander, Meunier, Camarasa, Lopez-Picado [19]. All calculation formulas require an estimation of the total blood volume of the patient. The formulas take into account height, weight, body surface area and gender of the patient. All blood loss estimation formulas showed a significant tendency to overestimate blood loss [6].

The aim of the study is to compare several methods of blood loss quantification—visual estimation by a surgeon and an anesthesiologist, gravimetric method, calculation method and the spectrophotometric method in real surgical settings.

There is still no gold standard in blood loss measurement, because accurate measurement of blood loss is difficult. The most accurate and reference methods are based on “hemoglobin extraction analysis” using spectrophotometry [6]. Visual estimation method was chosen because this method is most frequently used in a real surgical setting and it is the easiest method. Gravimetric method was chosen because it is a standard method to measure blood loss in certain surgical fields (gynecological surgery, orthopedic surgery, etc.).

We hypothesized that visual estimation, gravimetric measurement, and calculation method will significantly differ from measured hemoglobin loss by spectrophotometry.

## Methods

### Participants

The study was designed as a single-center prospective cohort study. All patients who were scheduled for elective HPB surgery were screened and assessed for eligibility. A total of 61 adults patients undergoing elective liver or pancreas surgery between May 2021 – June 2022 at Department of Surgery, University Hospital Hradec Kralove, Czech Republic were enrolled to the study. We decided for patients undergoing elective HPB surgery because expected estimated blood loss over 200 ml was needed for the statistical analysis [12]. The inclusion criteria were adult patients undergoing elective liver or pancreas surgery, age of patient ≥ 18 years, signed informed consent. The exclusion criteria were patient’s coagulation disorder (congenital or iatrogenic due to the chronic use of anticoagulants), use of cell saver suctioning during operation, clotting/damage blood samples, lack of compliance, informed consent not provided or refusal to participate.

The study was registered under the title " Blood Loss Quantification During Major Abdominal Surgery" at ClinicalTrials.gov with the registration number NCT05316649.

### Study variables

The basic clinical data of the 61 patients were collected, namely age, height, weight, body mass index (BMI), use of anti-coagulants, comorbidities, physical status (American Society of Anesthesiologists classification system score, ASA), operative time, perioperative catecholamine use, blood transfusions, fluids intake, volume of irrigation fluids, diuresis, hospital stay.

### Study outcome

Patients venous blood samples for blood count (including hemoglobin concentrations) were drawn before incision, and after surgery at 1, 24 and 48, 72 h, on the postoperative day 5 and at the end of hospital stay using an automated hematology analyzer XN-10 (Sysmex, Kobe, Japan).

The suction canister and surgical drapes are weighed before and after the surgical procedure with a Kern, PCB 6000–0 with an accuracy of ± 1 g (Balingen, Germany). Gravimetric blood loss (vGBL) is determined by assessing the weight difference after subtracting weight of added fluids [11]. Every gram of weight difference equivocal to 1 mL of blood loss [12].

The suction canister was heparinized before surgery (10,000 IU of heparin in 100 ml saline solution) to prevent clotting. The total volume contained in the canister was measured after the end of the surgical procedure by a system capable of determining differences up to ± 10 mL, weighted with a Kern, PCB 6000–0 with an accuracy of ± 1 g (Balingen, Germany) and analyzed for hemoglobin concentrations by spectroscopy using cell count in “body fluid” mode, which is more sensitive to lower cell counts in fluids.

The volume of irrigation fluids used during surgical procedure was carefully recorded as well as infusions, injections and transfusion volume. If necessary, vasoactive agents were titrated to obtain a mean blood pressure of > 65 mmHg during procedure.

Surgery time duration (skin to skin) was recorded. A daily visit of the study patients was made by clinical investigators or a delegated physician. All protocol-required information collected during the trial were entered into the patient’s record form.

Hemoglobin mass loss (hbMBL) for each case is calculated using the spectrophotometric measured hemoglobin concentration from the suction canister. This value is multiplied by the total volume of the suction canisters and the calculated fluid volume from surgical drapes.

To obtain total lost hemoglobin mass loss in g:

$$hbMBL=hemoglobin \,concetration \,from \,canister\,*\,(suction \,canister \,volume+fluid \,volume \,from \,surgical \,drapes)$$

where fluid volume from surgical drapes is calculated as:

$$fluid \,volume \,from \,surgical \,drapes=\frac{\left(suction \,canisters \,volume\right)\times \left(weight \,difference \,of \,surgical \,drapes \,before \,and \,after \,use\right)\,}{weight \,of \,suction \,canisters \,volume}$$

The spectrophotometric measured blood loss (vMBL) in mL is calculated using measured hemoglobin mass loss (hbMBL) and patient’s average pre- and postoperative hemoglobin, the vMBL is obtained:

$$vMBL=\frac{hbMBL \,(measured \,hemoglobin \,mass \,loss \,in \,g)}{mean \,\left(pre \,and \,postoperative\right) \,hemoglobin \,(in \,g/dL)}$$

For calculated blood loss (vCBL) was used López-Picado's formula [19] based on anthropometric and hematological parameters:

$$vCBL=\frac{\left[EBV\times \left(Hc{t}_{i}-Hc{t}_{f}\right)+transfused \,RBC \,volume\right]}{Hc{t}_{mean}}$$

where Hct_i_ (initial hematocrit) is the patient’s preoperative hematocrit, Hct_f_ (final hematocrit) is the patient’s postoperative hematocrit. Postoperative time point of the final hematocrit is not specified in the original formula, therefore in accordance with another studies (1) Hct_f_ in this trail is determined 48 h after surgery or when hematocrit reached the nadir level after operation. The transfused RBC volume is calculated as follows: 1 Unit packed homologous blood = 450 mL × hematocrit of the transfused blood; 1 Unit packed autologous blood = 450 mL × hematocrit in the pre-surgical anesthesia consultation. Hct_mean_ is the mean hematocrit (between Hct_i_ and Hct_f_). EBV is the estimated blood volume determined using the ICSH formula [18]:

- Female:

$$\mathrm{EBV }({\text{mL}})=\mathrm{Plasma \,volume }\,({\text{mL}})+\mathrm{red \,cell \,volume }({\text{mL}})=[\mathrm{weight }{\left({\text{kg}}\right)}^{\mathrm{0,425}}\times \mathrm{height }{({\text{cm}})}^{\mathrm{0,725}}]\times \mathrm{0,007184}\times \mathrm{2,217}+\mathrm{age }\,({\text{years}})\times \mathrm{1,06}$$

b) Male:

$$\mathrm{EBV }\left({\text{mL}}\right)=\mathrm{Plasma \,volume }\,\left({\text{mL}}\right)+\mathrm{red \,cell \,volume }\,\left({\text{mL}}\right)=\left[\mathrm{weight }{\left({\text{kg}}\right)}^{\mathrm{0,425}}\times \mathrm{height }{\left({\text{cm}}\right)}^{\mathrm{0,725}}\right]\times \mathrm{0,007184}\times \mathrm{3,064}-825$$

At the end of the operation, a blood loss estimate was obtained from surgeon (sEBL) and anesthesiologist (aEBL).

### Statistical analysis

Statistical analyses were performed using NCSS statistical software (NCSS, Kaysville, UT, USA). Categorical data are expressed as absolute and relative frequencies. Quantitative data are expressed as means and standard deviations if normally distributed or medians and interquartile ranges in case of unnormal distributed data. Normality was tested with the Shapiro–Wilk test.

In pairwise comparison of methods, when normality was not rejected, the paired t-test was used. When normality was rejected the non-parametric Wilcoxon test was used. A *P*-value < 0,05 was considered statistically significant. Bland–Altman analysis with 95% confidence interval was used for the degree of agreement between the spectrophotometry method and other methods.

### Sample size calculation

The sample size calculation was based on the data from a previous study [9]. Power calculations revealed that a sample size of 54 pairs would be needed to detect a twofold difference between two methods with 95 percent power to detect the mean difference of 100 mL between these two methods. With an expected dropout rate of 10%, we planned to enroll 60 patients into the study.

## Results

Participants' data on age, gender, height, weight, BMI, physical status, surgical intervention, estimated blood volume, operative time, peroperative catecholamine use, blood transfusion, fluids intake, diuresis, hospital stay, participant preoperative and minimal postoperative hemoglobin level as well as the outcome measurement data from suction and surgical drapes and other methods than spectrophotometric measurement are summarized in the Table 1. Twenty-nine patients received pancreatic resection, twenty-seven patients received liver resection and five patients had different procedure (bile duct resection, biliary reconstruction, etc.). We found no differences between pancreatic resection and liver resection regarding outcome data from suction and surgical drapes or blood loss according to the method used. Only 3 patients received perioperative blood transfusion (4,91%). Perioperative catecholamines were used in 50.8% of the procedures. Median hospital stay was 11 days (interquartile range 7–15 days).

**Table 1:** Descriptive statistics

**Age** (yrs), mean (range)	66.7 (27–89)
**Gender**	
** Male**, frequency (%)	29 (47.5%)
**Female**, frequency (%)	32 (52.5%)
** Total**, frequency (%)	61 (100%)
**Height** (cm), mean (SD)	170.9 (8.92)
**Weight** (kg), mean (SD)	78.2 (15.67)
**BMI** (kg/m2), mean (SD)	26.7 (4.59)
**ASA physical status classification**, **frequency (%)**	
1	12 (19.7%)
2	32 (52.5%)
3	17 (27.8%)
**Estimated blood volume** (mL) (ICSH formula), median (IQR)	4548 (3974–5359)
**Surgical intervention, frequency (%)**	
Proximal pancreatectomy	22 (36.1%)
Distal pancreatectomy	7 (11.4%)
Minor liver resections	22 (36.1%)
Major liver resections	5 (8.2%)
Other procedures	5 (8.2%)
**Operative time (min), mean (SD)**	200.9 (73.79)
**Peroperative**	
Catecholamines use, yes/no, n	31/30
Blood transfusion, yes/no, n	3/58
Infusions (ml), median (IQR)	2400 (1600–350)
Diuresis (ml), median (IQR)	400 (200–775)
**Participants’ perioperative hemoglobin concentrations**	
Preoperative hemoglobin level (g/L), mean (SD)	117.4 (19.19)
Postoperative minimal hemoglobin level (g/L), mean (SD)	92.9 (14.11)
**Hospital stay** (days), median (IQR)	11 (7–15)
**Outcome data from suction and surgical drapes**	
Suction volume (mL), median (IQR)	450 (275–700)
Suction weight (g), median (IQR)	709 (543–982)
Suction hemoglobin level (g/dL), median (IQR)	34 (18–39)
Surgical drapes weight (g), median (IQR)	1448 (991–1774)
Number of surgical drapes used, median (IQR)	20 (14–30)
**Blood loss according used method**	
Spectrophotometric measurement (vMBL) (ml), median (IQR)	251 (109–411)
Estimated blood loss by surgeon (sEBL) (ml), median (IQR)	300 (200–500)
Estimated blood loss by anestesiologist (aEBL) (ml), median (IQR)	400 (225–700)
Calculation method (vCBL) (ml), mean (SD)	995 (602.2)
Gravimetric method (vGBL) (ml), median	1298 (822–1728)

*ASA* American Society of Anesthesiologist physical status classification, *BMI* body mass index, *ICSH* International Council for Standardization in Haematology, *IQR* interquartile range, *SD* standard deviation

While the mean preoperative hemoglobin concentration of the participant was 117.4 g/L (SD 19.9), the medium hemoglobin level from suction was 34 g/L (IQR: 18–39). Median of measured blood loss by spectrophotometry (vMBL) was 251 mL (IQR: 109–411). In all cases, statistically significant difference was found between spectrophotometric measurement and other methods (*p* < 0.05) as shown in the Table 2.

**Table 2:** Pairwise comparison of the methods of blood loss quantification

Pairwise comparison **Spectrophotometric measurement** (vMBL) with	Test	*p*- value
**Estimated blood loss by surgeon** (sEBL)	Paired-Sample T-Test T-value 2.2708	0.027
**Estimated blood loss by anestesiologist** (aEBL)	Paired-Sample T-Test T-value 5.5879	< 0.0001
**Gravimetric blood loss** (vGBL)	Wilcoxon Signed-Rank Test Z- value 6.7877	< 0.0001
**Calculation method** (vCBL)	Wilcoxon Signed-Rank Test Z- value 6.5651	< 0.0001

The Bland–Altman analysis of difference of Spectrophotometric measurement (vMBL) compared to other methods of estimated blood loss is summarized in Table 3. The Bland–Altman analysis of difference of Spectrophotometric measurement (vMBL) – Estimated blood loss by surgeons (sEBL) resulted in a bias -68.7 ml (95% confidence interval [CI]: -129.3–-8.2), a lower limit of agreement of -531.9 ml (95% CI: -636.0–-427.9) and an upper limit of agreement of 394.5 ml (95% CI: 290.5–498.6). The Bland–Altman analysis of difference of Spectrophotometric measurement (vMBL) – Estimated blood loss by anesthesiologists (aEBL) resulted in a bias -206.6 ml (95% confidence interval [CI]: -280.5–-132.6), a lower limit of agreement of -772.6 ml (95% CI: -899.7–-645.5) and an upper limit of agreement of 359.4 ml (95% CI: 232.3–486.5). The Bland–Altman analysis of difference of Spectrophotometric measurement (vMBL) – Gravimetric blood loss (vGBL) resulted in a bias -1055.6 ml (95% confidence interval [CI]: -1191.5–-919.8), a lower limit of agreement of -2095.4 ml (95% CI: -2328.9–-1861.9) and an upper limit of agreement of -15.8 ml (95% CI: -249.3–217.6). The Bland–Altman analysis of difference of Spectrophotometric measurement (vMBL) – Calculation method (vCBL) resulted in a bias –675.4 ml (95% confidence interval [CI]: -814.8–-535.9), a lower limit of agreement of -1742.2 ml (95% CI: -1981.8–-1502.7) and an upper limit of agreement of 391.4 ml (95% CI: 151.9–630.9).

**Table 3:** Bland–Altman analysis of the methods

Bland–Altman analysis of **Spectrophotometry** and	Difference (mL)	95% Limits of agreements	*p*-value
**Estimated blood loss by surgeon (**sEBL)	-69	(-532, 395)	0.12
**Estimated blood loss by anesthesiologist** (aEBL)	-207	(-773, 359)	0.20
**Calculation method** (vCBL)	-675	(-1742, 391)	0.022
**Gravimetric method** (vGBL)	-1056	(-2095, -16)	0.002

**Fig. 1:**
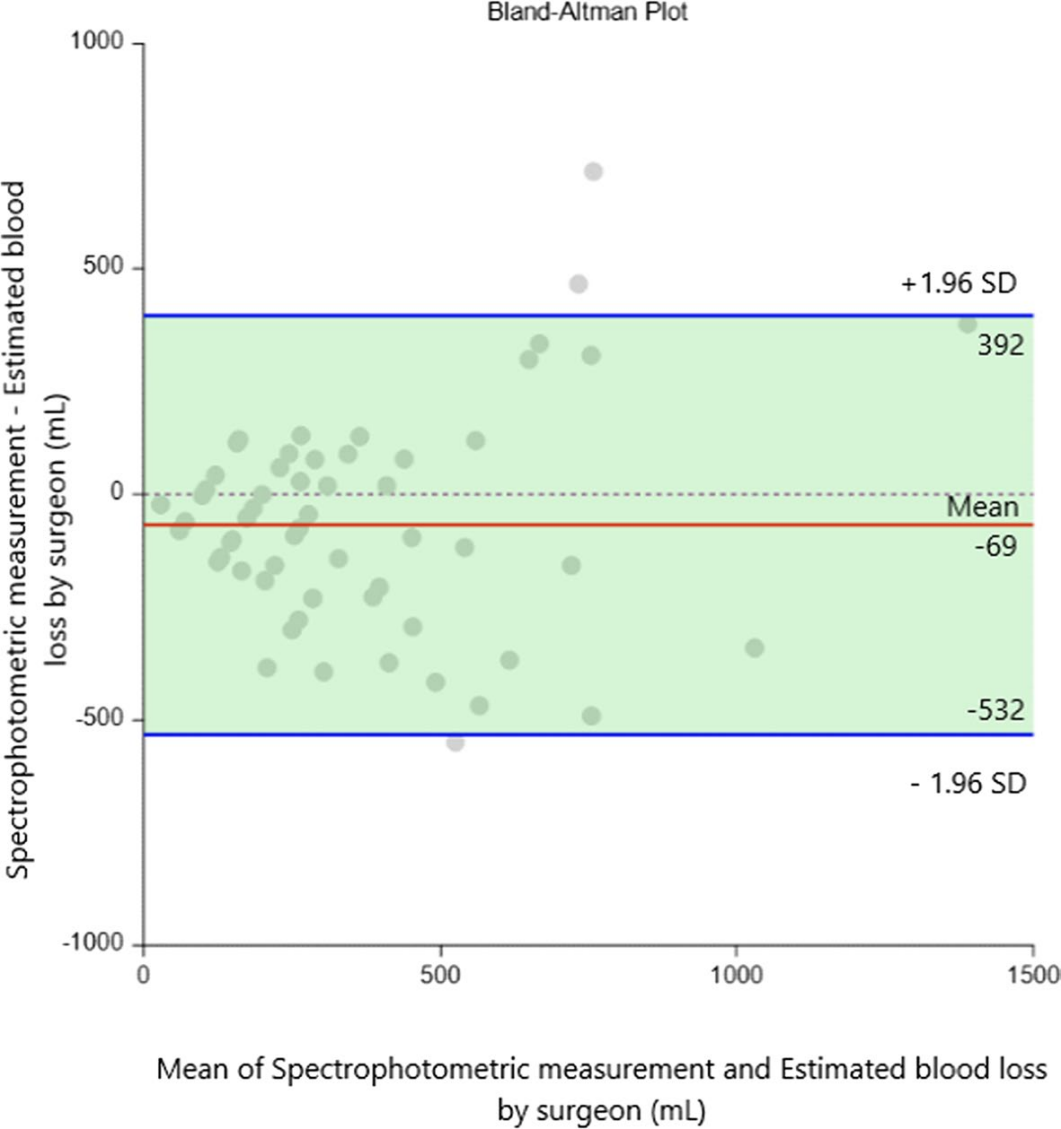
Bland–Altman plot of differences between Spectrophotometric measurement and Estimated blood loss by surgeon (sEBL). In the Bland–Altman plot, bias is represented by a solid red line, 95% limits of agreement are indicated by solid blue lines

All methods overestimated spectrophotometric measurement of blood loss as shown Table 3. The surgeons' estimate was the closest to the spectrophotometric measurement. Estimated blood loss by surgeon differ from spectrophotometric measurement less than ± 463mL with 95% probability as shown in the Fig. 1.

## Discussion

There are several methods for blood loss quantification in surgical procedures. In this study, we compared all the methods in a real surgical setting. We hypothesized that visual estimation by surgeons and anesthesiologists, as well as gravimetric measurement and calculation method would be significantly different from the most accurate method, e.g. measured hemoglobin loss by spectrophotometry (*p* < 0.05). All the methods overestimated the blood loss compared to the accurately measured blood loss by spectrophotometry.

Visual estimation of blood loss is still the standard method of choice in most institutions [8]. Even though the visual estimation by surgeon in this study was the closest to the measured hemoglobin loss by spectrophotometry; -68.7 ml (95% confidence interval [CI]: -129.3–-8.2), the surgeons' estimate also shows significant variations. Irrigation fluids, lymph, bile, serum, ascites, urine, and other fluids often combine with lost blood, but do not alter its appearance to an extent that is typically appreciable visually, which can affect estimated blood loss [9]. We proved that the visual estimation of blood loss is unreliable and inaccurate. No specialty, level of experience, or self-assessment of ability is associated with improved estimation [10]. We did not confirm the commonly accepted assumption that the surgeons underestimate blood loss and the anesthesiologists overestimate it [13]. In our study, even surgeons slightly overestimated blood loss, however, not as much as the anesthesiologists.

This study confirms that one of the oldest methods of quantifying blood loss gravimetric method described by Wangensteen [11] is easy but neither precise nor accurate [13], especially with increased dilution by rinsing fluid [14, 15]. The mathematical approach to evaluate blood loss is based on anthropometric and hematological parameters [19]. It is important to note that all blood loss estimation formulas showed a significant tendency to overestimate blood loss [6]. Although this method of measuring blood loss eliminates the need for perioperative weighing, measuring surgical drapes and canisters, the results are significantly inaccurate compared to the spectrophotometric measurements, as we demonstrate in this study using the Lopez-Picado formula.

It is very important to quantify the blood loss more accurately because blood loss is an important parameter of quality of the surgical procedure. There are numerous studies which corelate blood loss with postoperative morbidity or mortality. However, with inaccurate blood loss estimate, unreliable findings can be reached. Blood loss quantification also play an important role in blood transfusion decisions. Inaccurate blood loss estimate can cause unnecessary blood transfusion application. Inappropriate transfusion of blood products is associated with increased risks and negatively influences patient´s outcome and long-term survival in oncological patients.

Similar results were reached by Perri et al. [20]. The authors performed a systematic review of original studies published between 2006 and 2021 reporting the blood loss in patients undergoing pancreatic or hepatic resection. The authors concluded that standardization of intraoperative blood loss quantification is urgently needed in HPB surgery to ensure the consistency of reporting the results of blood loss comparison with other factors [20].

The main limitation of this study is that fluid volume from surgical drapes was indirectly calculated through the weight before and after use and not directly measured. It is possible that the concentration of hemoglobin in surgical drapes is slightly different from the concentration of hemoglobin in suction canister. In our study, we assumed that these concentrations are alike, as it was shown in the study by Thomas et al. [9]. However, it would be more accurate if hemoglobin losses from surgical drapes were measured directly separately. Nevertheless, it would make the measurement methodology even more difficult in real surgical conditions.

## Conclusion

In agreement with previous studies [6, 8–10, 13] we conclude that the assessment of blood loss using surgeon and anesthesiologist estimates, gravimetric methods, and calculation methods are all significantly inaccurate. All studies which use simple blood loss estimate and corelate it with other parameters should take it into account. We did not confirm the commonly accepted assumption that the surgeons underestimate blood loss and the anesthesiologists overestimate it [9]. In our study, even surgeons slightly overestimated blood loss. Surprisingly, this study showed that calculation method and gravimetric method are even less accurate than simple surgeon’s visual estimate.

Spectrophotometric measurement of blood loss in real surgical setting is difficult, and is not suitable for routine use due to the economic or methodological reasons. Therefore, despite inaccuracy, surgeon's estimation is very likely to remain the most used method of blood loss in most departments.

## Data Availability

The materials described in the manuscript, including all relevant data, are freely available to any scientist wishing to use them for non-commercial purposes, without breaching participant confidentiality. The datasets used and analyzed during the current study are available from the corresponding author on reasonable request.

## Abbreviations

aEBL: Estimated blood loss by anesthesiologist in volume units (mL)
EBV: Estimated blood volume
hbMBL: Measured hemoglobin mass loss in mass units (g)
Hct_i_: Initial hematocrit, is the patient’s preoperative hematocrit
Hct_f_: Final hematocrit
Hct_mean_: mean hematocrit (between Hcti and Hctf)
HPB: Hepato-pancreato-biliary (surgery)
ICSH: International Council for Standardization in Haematology
RBC: Red blood cell
sEBL: Estimated blood loss by surgeon in volume units (mL)
vCBL: Calculated blood loos in volume units (mL)
vGBL: Gravimetric blood loss in volume units (mL)
vMBL: Measured blood loss in volume units (mL)
